# Attention Does Not Affect the Speed of Subjective Time, but Whether Temporal Information Guides Performance: A Large‐Scale Study of Intrinsically Motivated Timers in a Real‐Time Strategy Game

**DOI:** 10.1111/cogs.12939

**Published:** 2021-03-23

**Authors:** Robbert van der Mijn, Hedderik van Rijn

**Affiliations:** ^1^ Department of Experimental Psychology University of Groningen

**Keywords:** Time perception, Interval timing, Attention, Attentional gate, StarCraft2, Video game telemetry

## Abstract

Many prepared actions have to be withheld for a certain amount of time in order to have the most beneficial outcome. Therefore, keeping track of time accurately is vital to using temporal regularities in our environment. Traditional theories assume that time is tracked by means of a clock and an “attentional gate” (AG) that modulates subjective time if not enough attentional resources are directed toward the temporal process. According to the AG theory, the moment of distraction does not have an influence on the subjective modulation. Here, we show, based on an analysis of 28,354 datasets, that highly motivated players of the online multiplayer real‐time strategy game StarCraft2 indeed respond later to timed events when they are distracted by other tasks during the interval. However, transient periods of distraction during the interval influence the response time to a lesser degree than distraction just before the required response. We extend the work of Taatgen, van Rijn, and Anderson (2007) and propose an alternative active check theory that postulates that distracted attention prevents people from checking their internal clock; we demonstrate that this account better predicts variance observed in response time. By analyzing StarCraft2 data, we assessed the role of attention in a naturalistic setting that more directly generalizes to real‐world settings than typical laboratory studies.

## 1 Introduction

Responding to our environment at the right moment often provides an advantage over too early or too late responses. However, when we only start preparing at the moment when a response is needed, the response will be too late. Thus, we have to start the preparation before the expected response is needed, taking into account the duration of the response execution. However, as the preparation of a response is a noisy process, we should aim to be prepared before our action is actually needed. As a consequence, we often face the situation in which we are prepared to respond, but have to withhold the execution of the action for a certain amount of time. For example, an experienced cook prepares to remove a pan from the stove after the potatoes are done, but before they are overcooked; and an athlete prepares to be ready to go at exactly the moment of the expected go‐signal, but definitely not earlier to prevent disqualification. Whereas the athlete is fully focused on keeping track of time for one important event, the cook might have several pans on the stove which all need attention. When we have to pay attention to several tasks at once, we might lose track of time for one of the tasks, which might cause us to miss the onset altogether. Here we will present a study using a real‐time strategy (RTS) game, in which sharing attention between timing and other tasks is critical for optimal game performance, that can shed light on the mechanisms through which attention acts on time perception.

The mechanisms that allow humans and animals to keep track of time are described in theories that are typically supported by carefully designed laboratory experiments. Most of these theories assume some sort of timekeeping mechanism that interacts with memory to keep track of previously encountered durations (Buhusi & Meck, 2005; Gibbon, Church, & Meck, 1984; Grondin, 2010; Zakay & Block, 2004). However, a great amount of experimental control is required to investigate how time is being kept, often resulting in experimental tasks that lack ecological validity (Darlow, Dylman, Gheorghiu, & Matthews, 2013; Matthews & Meck, 2014; Schlichting et al., 2018). For example, interval reproduction tasks typically entail the detection of a sound or visual stimulus with a clear onset and offset. Such clear markers of an event are rare in the real world and may not generalize to ecologically valid timing situations. Therefore, some studies have explicitly targeted better generalization of the experimental paradigms (Matthews & Meck, 2014; Moon & Anderson, 2013; Schlichting et al., 2018; van Rijn, 2014; Zakay & Shub, 1998). For example, Schlichting et al. (2018) demonstrated that even when more realistic stimuli were used that did not provide clear on‐ and offset signals, classical findings as the central tendency effect (Vierordt, 1868) and scalar property (Gibbon, 1977; Staddon, 2005) were retained. However, many aspects of the task were still rather artificial. For example, the timing task was always the primary focus of the experiment, whereas timing in the real world occurs in a more dynamic context in which attention is often divided over many tasks.

The most prominent class of theories of interval timing assumes an internal time source, often referred to as a pacemaker, that emits pulses at a predictable rate. By accumulating these pulses during an interval and memorizing the number of pulses accumulated, a decision can be made as to whether the amount of elapsed time since the start of the interval is longer or shorter than a previously experienced duration (Buhusi & Meck, 2005; Gibbon et al., 1984; Wearden, 1991). Several temporal illusions, such as *“*time flies when you’re having fun” or “that boring lecture seemed to last forever!” can be explained by this pacemaker‐accumulator (PA) mechanism. For example, if the pacemaker is temporarily sped up, it emits more pulses in the same amount of time. If a specific interval learned under normal pacemaker conditions results in *n* pulses, the same interval will, with a clock sped up with factor *x*, be associated with n*x pulses, and thus this interval will subjectively feel to have lasted longer. Although the neural mechanism of this pacemaker component is still under discussion, studies have demonstrated that the speed of the clock is influenced by emotion (Halbertsma & Van Rijn, 2016; Lui, Penney, & Schirmer, 2011), arousal (Gil & Droit‐Volet, 2012), and even temperature (Maanen et al., 2019; Wearden & Penton‐Voak, 1995). However, speeding up or slowing down of the pacemaker mechanism is not the only factor leading to time being perceived as passing slower of faster. If the speed of the pacemaker stays constant, but not all emitted pulses reach the accumulator, a smaller number of pulses is accumulated in the same duration, which results in a subjective shortening of the duration of that interval. This explanation plays an important role in certain PA theories that assume a central role of attention in temporal processing (Lejeune, 1998; Zakay & Block, 1997).

More specifically, the attentional gate (AG) theory (Zakay & Block, 1997) assumes that pulses must pass through an AG that prevents pulses from reaching the accumulator if not enough attention is allocated to time. Consequently, when nontemporal attentional load is higher during the reproduction than during the earlier encounters of that interval, the objective reproduced duration will be longer as it will take longer for the same number of pulses to reach the accumulator due to the partially closed “gate.” The AG theory has found empirical support in a series of studies (Block, Hancock, & Zakay, 2010; Lejeune, 1998). Participants in these studies systematically overreproduced learned intervals if temporal information processing had to compete for attention with other tasks.

Although the idea of an AG being the cause of subjective time to slow down when attention is distracted has found substantial support, alternative explanations cannot be ruled out. This was shown in a study by Taatgen, van Rijn, and Anderson (2007) in which participants, in separate blocks performed either a simple or more difficult task. Alongside the main, nontemporal task, participants were given the option to increase their score by simultaneously reproducing an interval. The AG theory would predict that if a participant switched from the easy to the more difficult block, the reproduced durations should increase. However, no such shift was observed. Instead, participants seemed to either be relatively accurate or just omit the response to the timing task in the difficult block. This indicates that instead of an effective “slowing of the clock,” the role of attention might be better conceptualized as “forgetting to respond in time.” The current study further explores this notion, which we refer to as the active check (AC) theory.

However, following the introductory paragraph, a concern about studies investigating the role of attention on timing is their use of artificial tasks and stimuli. A study that is often quoted as a notable exception is the work by Zakay and Shub (1998) in which fighter pilots were asked to time a 14‐s interval while engaging in a flight simulation of varying degrees of difficulty. The pilots more frequently overestimated or omitted their responses to the timing task when performing more difficult simulated flights. However, it is important to note that the imposed timing task was completely unrelated to the real‐world context, as the timing task was completely isolated from the simulated flight. This is in stark contrast with real‐world timing, where timing accuracy is often critical to the outcome of an associated task. A paradigm in which timing was more central to task performance was explored by Moon and Anderson (2013). They asked participants to play a fast‐paced computer game in which a quick execution of well‐timed actions such as pressing an action‐key was necessary. However, this paradigm focused on temporal planning at the intersection of motor preparation (or motor timing) and the timing of longer intervals (Buhusi & Meck, 2005). Additionally, no major secondary tasks had to be executed during the execution of the timed actions. To our knowledge, there are no studies in which the influence of shared attention is evaluated on performance on tasks in which the participant is truly invested to time accurately, yet not explicitly instructed to do so.

Here we use video game telemetry (Huang, Yan, Cheung, Nagappan, & Zimmermann, 2017; Thompson, McColeman, Stepanova, & Blair, 2017; Yan, Huang, & Cheung, 2015) of StarCraft2 (Blizzard Entertainment, 2013), an RTS game. Like most RTS multiplayer games, the objective of StarCraft2 is to defeat an opponent by destroying their virtual buildings and units. Successful players quickly expand their initial base by strategically timing the construction of new buildings, units, and upgrades to gain an advantage over their opponent(s). Earlier work focused on motor skill learning in an ecologically valid setting by analyzing player performance in StarCraft2 (Thompson et al., 2017). An important characteristic of StarCraft2 is its time‐critical nature: A player’s success hinges on the accurate timing of in‐game actions as players should utilize temporal regularities to optimize their responses. To optimally execute the primary task (i.e., winning the game), estimation of these regularities is an implicit but important subtask while other processes are executed. And thus, according to the AG theory, performance on the time‐critical sub‐tasks should suffer as a function of the player’s attention toward timing.

**Fig. 1:**
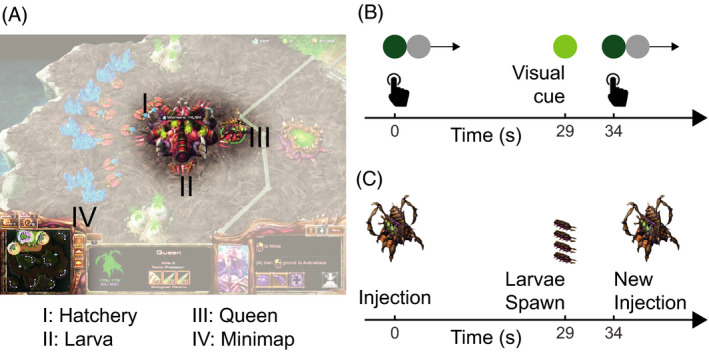
Screenshot of StarCraft2 (A) and a schematic description of a timing task in which a participant is asked to initiate a new interval as soon as possible after an earlier interval has ended (B) and Queen Injection task (C). In this example, the player has executed a new injection (at second 34) 5 s after the larvae from the previous injection (at second 29) spawned.

StarCraft2 provides an opportunity to study truly motivated timers and how being distracted influences their timing performance. In the current study, we will assess the influence of shared attention on timing in StarCraft2. We will focus on one specific action called an “injection,” which is performed on a “hatchery” (see Fig. 1). Every 11 s, hatcheries spawn virtual resources (i.e., “larvae”) that the player can use to defeat the opponent. If a hatchery has been injected by a special unit (a “queen”) and 29 s have passed, the hatchery will spawn additional larvae. During the 29 s, no additional injections can be made. It is therefore in the player’s interest to time these injections well and thereby minimize the lost time between a spawning of the additional larvae and a new injection. Yet the player will need to execute other actions while keeping track of the 29 s in order to optimize resource harvesting. It is important to note optimal performance will be obtained when a player is ready to execute the action just after the 29 s. Therefore, players should try to balance the amount of attentional resources between keeping track of the interval to prevent “hatchery idle‐time” and keeping track of other tasks to prevent idle time of the player himself.

Optimizing timed injections can be seen as a real‐world analog of a typical laboratory temporal reproduction task—illustrated in Fig. 1B—in which participants are instructed to press a reward circle as often as possible. However, after each press the reward circle will be deactivated for 29 s. To maximize earnings, participants must be as fast as possible. Although in the Queen Injection task (Fig. 1C) the clock does not reset when a player is too early, moving the camera and checking the hatcheries too early does take time that could have better been spent on other tasks. These tasks entail monitoring the different parts of the playing field, predicting enemy moves, and managing their own units. Professional StarCraft2 players sometimes perform over 5 actions per second (APS), but they are still required to perform their injection every 29 s. Following the AG theory, this would mean that if a high number of other attention‐requiring actions are performed by a player, the latency of their injections should increase. That is, if we assume that the response time of injections is a function of temporal expectation, then increased APS should drive an increased response time.

The AG and AC theories both predict that a high level of APS at the spawning moment will cause longer delays between the moment of spawning and the next injection. However, they make different predictions regarding the effects of transient phases of high APS *during* the 29‐s injection delay. According to the AG theory, mean APS during the entire 29‐s period will impact the response time as diverting attention at any time during the delay will affect the number of accumulated pulses. According to our AC theory, APS *during* the 29‐s interval should have no effect on response time. Rather, response time would only be influenced by APS around the time of responding. These differences between the AC and AG theories yield distinctive behavioral signatures, which can be empirically tested.

We present a study where a large set of real‐world data is used to test these hypotheses about the interaction between timing and attention. For the first time, we use video game telemetry to explore human interval timing in an ecologically valid setting in which participants are intrinsically motivated to accurately time.

## 2 Methods

**Fig. 2:**
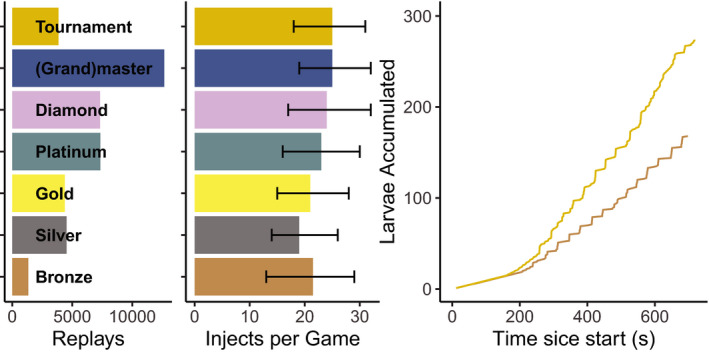
(A) Player skill is represented by their league. (B) Over the course of a game players perform roughly between 15 and 30 injections. (C) The difference in accumulation of larvae between two typical games (by a Bronze and a Tournament league player) illustrates how Queen injects are more consistent for the high‐level player compared to the low‐level player.

We collected 28,354 replay files of StarCraft2 games that were played between May 2013 and July 2016 from www.spawningtool.com. All games were played in the *Heart of the Swarm* version of the game. Replay files were made public by uploading to Spawningtool by individual players. Analysis of this data was monitored and approved by the Ethics Committee Psychology of the University of Groningen. Analysis scripts and statements about data files are accessible via https://osf.io/fwmj7/. Each replay contains data of one or more players. Replay files were parsed using sc2reader (Kim, 2011) and custom python scripts; 10 files were too damaged to be recovered and thus excluded. The parser identifies keystrokes and mouse actions of the players to deduce game states, actions, and timings. Data used in this manuscript consisted of the replay files that met the following criteria: One or multiple players selected Zerg as race (the injection game option is only available when a player chose to play as Zerg), the game was longer than 3 minutes and shorter than 30 minutes, and the replay file contains information about the *league* of the players (i.e., skill). If a player had played in multiple leagues, their rounded mean league was used in the analysis. The distribution of included players over the leagues is displayed in Fig. 2A. Additionally, we excluded replays in which less than three injections were recorded (7.7% of the included replays).

Like most RTS multiplayer games, the objective of StarCraft2 is to defeat an opponent by destroying their virtual buildings and units. Successful players can quickly expand their initial base by strategically timing the construction of new buildings, units, and upgrades to gain an advantage over their opponent(s). *Larvae* are the primary units for players who opt to play as Zerg. Larvae spawn every 11 s at each of the player’s hatcheries. The game starts with a single hatchery for a Zerg player, but throughout the game players typically construct additional hatcheries to increase the rate at which larvae become available. To construct a hatchery, or any Zerg building or unit, a player needs to invest a larva and additional resources. Additionally, Zerg players may construct *queens* that can use their *spawn larvae* ability to inject a hatchery.

Although the game requires a player to attend to many tasks simultaneously, only the timing of the spawn larvae ability, called an *injection*, is analyzed in this study. This ability can only be performed if at least 29 s have passed since the previous injection. After the 29 s have passed, the player is granted four extra larvae. Thus, perfectly timed injections increase the default larvae‐spawn rate by a factor of 2.5. To execute an injection, the player must (a) move the focus of the game to a hatchery by either clicking near it on the mini‐map or moving to it manually using the arrow keys; (b) selecting the queen near the hatchery; (c) selecting the spawn larvae ability of the queen; and (d) clicking the hatchery. Most players use a combination of customizable hotkeys to do steps (a)–(c) to increase their speed. After the hatchery is clicked, an empty bar appears across the width of the hatchery, filling up over the course of the next 29 s. However, as soon as the focus of the game is moved, this bar is not visible anymore. The typical accumulation of larvae during the course of a game is displayed in Fig. 2B.

Timing accuracy was determined by injection response time (IRT). IRT is defined by the amount of time passed between the moment the larvae from a previous injection are spawned and the moment a new injection is performed, which is referred to by StarCraft2 players as “missed injection.” A player typically has multiple hatcheries, some of which have a queen nearby with the purpose of injecting that hatchery. IRTs that deviated more than three absolute median deviations from the median were not considered in the analysis (13.1%). The distribution of number of injections per game is shown in Fig. 2C.

As an index of the attention allocated to other parts of the game, we calculated the number of actions performed per second. This APS measure was calculated based on three actions: a unit or group of units was selected using the cursor; a group of units was selected using a previously assigned hotkey; a command was issued. APS was calculated during two periods in relation to an injection: the 5 s leading up to the injection (APS Inject), and the 5‐s interval starting 10 s before the spawning of larvae from the previous injection (APS During, i.e., from second 19 to 24 in Fig. 1C). Note that this definition prevents these APS periods to overlap, even if the player would inject immediately after the additional larvae spawned. Although Actions Per *Minute* is the standard measure to express action frequency in the gaming community (e.g., Lewis, Trinh, & Kirsh, 2011; Thompson, Blair, Chen, & Henrey, 2013), we will report APS as the time windows relevant in the current work cover multiple seconds, rather than minutes.

### 2.1 Analysis

We performed hierarchical mixed linear effects regression using the *lme4* package in R (Bates, Mächler, Bolker, & Walker, 2015). The heavy‐tailed distribution of IRT was transformed using the Lambert W function (Goerg, 2012; Grabot et al., 2019). The transformed variable will be referred to as IRT_W_. IRT_W_ was used as the continuous dependent variable in all models. Variables were added to an empty regression model; resulting models were judged based on their Bayes information criterion (BIC). The model with the lowest BIC was considered only if the Bayes Factor (BF) of that model over a previous model was larger than 3 (Raftery, 1995). The BF of an alternative hypothesis over a null hypothesis was calculated by ${\text{BF}}_{01}=\exp\left(\frac{\Delta {\text{BIC}}_{10}}{2}\right)$ where $\Delta {\text{BIC}}_{10}$ is the difference in BIC of the two models (Wagenmakers, 2007).

First, we added Played Id and Game Id as random effects. Second, exploratory plots revealed League and Game Stage to be strong predictors of IRT. To control for the effect of skill on IRT, league of the player was considered as an unweighted effect‐coded fixed factor. And as additional resources are more critical in the initial stages of the game than in later stages, we accounted for the effects of game stage by including the relative game stage (i.e., the time since the start of the game divided by the total game duration) to the model. Finally, our main hypothesis was tested by adding APS Inject and APS During to the model separately as continuous fixed factors. To summarize the heavy tailed IRTs in the same figures as the estimated models, the median IRT per game was calculated. Five quantiles of these medians—20%, 40%, 50%, 60%, and 80%—are displayed together with model predictions and are referred to as *summary quantiles*.

## 3 Results

**Fig. 3:**
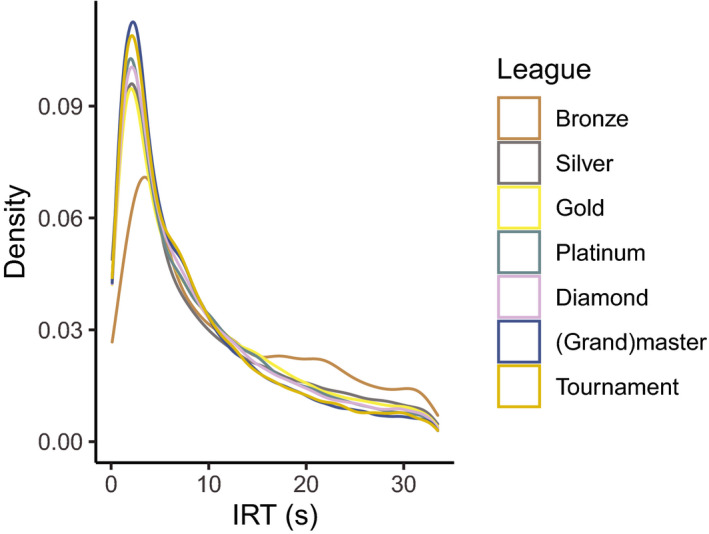
Distribution of IRT per league.

After applying the inclusion criteria to the parsed replay files, observations of 13,456 games played by 3,625 players remained. The distribution of IRT per league is shown in Fig. 3. Since the raw IRTs were heavy tailed, the Lambert W transformed IRT_W_ was used in further analysis. When presenting the model prediction in figures, IRT_W_ is transformed back using the function $\text{IRT}={\text{IRT}}_{W}*\exp\left({\text{IRT}}_{W}\right)$.

**Fig. 4:**
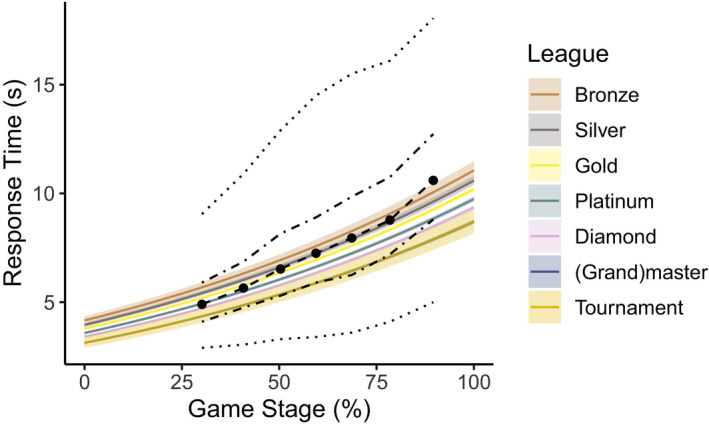
Model estimation (in color) of IRT per league over the course of a game. The 20%, 40%, 50%, 60%, and 80% summary quantiles of the empirical data are plotted by dashed and dotted lines, clustered at seven quantiles of Game Stage.

We set out to assess whether IRT_W_ depends on APS During and APS Inject. Fig. 4 shows the *summary quantiles* of IRT across seven quantiles of Game Stage, pooled over league. IRT becomes slower as the game progresses. Furthermore, Fig. 4 shows model predictions of the effect of Game Stage on IRT per League. First, random factors were selected by comparing an empty linear model predicting IRT_W_ (*BIC* = 530,975) to three models with either Player ID (*BIC* = 524,115), Game ID (*BIC* = 524,252), or both (*BIC* = 521,894, *BF_10_ > *1,000). As including both random effects yielded the lowest BIC, we compared subsequent models to this model. Next, we assessed Game Stage. The model that included Game Stage as a fixed effect with random slopes of Game Stage on Game ID (*BIC* = 507,912, *BF_10_ > *1,000) was preferred over the ones that included random slopes of Player ID (*BIC* = 509,971), both random slopes (*BIC* = 508,135), and no random slopes (*BIC* = 509,677). Adding the unweighted effects coded variables for League as fixed main effects improved the model further (*BIC* = 507,879, *BF_10_* > 1,000), but its interaction with Game Stage did not (*BIC* = 508,015). Fig. 4 shows the model estimation of IRT per league over the course of a game. IRTs become slower as the game progresses similarly in all leagues. In addition, IRT decreases as the League of the player increases.

**Fig. 5:**
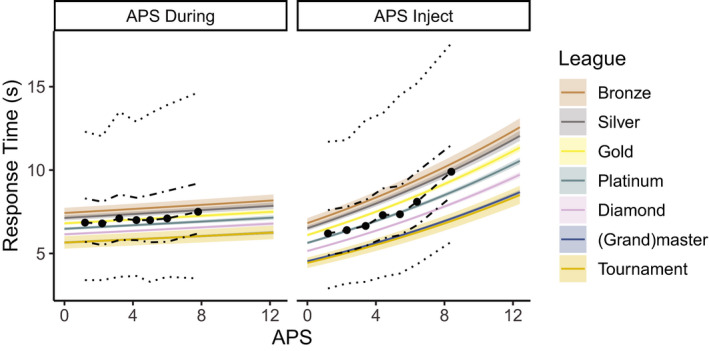
Model predictions or IRT per league over the two APS variables. The summary quantiles across the seven quantiles of Game Stage are represented by the dashed and dotted lines.

Adding either APS During or APS Inject to the model as continuous fixed factor resulted in improved models (both *BF_10_* > 1,000). Fig. 5 shows the *summary quantiles* of IRT across seven quantiles of both APS variables separately pooled over League. The steeper lines for APS Inject than for APS During already suggest that the APS just before the inject is a better predictor of the IRT than the action per second during the last inject phase, a finding confirmed by the analyses. APS Inject improved the model when considered as just the main effect (*BIC* = 504,598, *BF_10_* > 1,000). This model was preferred over models with an interaction with Game Stage (*BIC* = 504,619), an interaction with League (*BIC* = 504,647), and an interaction with both Game Stage and League (*BIC* = 504,667). However, APS During also improved the model when it was entered as just the main effect (*BIC* = 507,801). This model was preferred over models with an interaction with Game Stage (*BIC* = 507,828), an interaction with League (*BIC* = 507,906), and an interaction with both Game Stage and League (*BIC* = 507,934). Although both APS variables improved the model when considered separately, a full model including both APS variables without interaction (*BIC* = 504,613) was only preferred over the model with APS During (*BF_10_* > 1,000), but not over the model with APS Injects (*BF_10_* < 0.001). This suggests that the variance explained by APS During is partially explained by APS Inject.

## 4 Discussion

We set out to address how timing is influenced by attention in naturalistic settings. Using a large set of naturalistic data from the RTS game StarCraft2, we show that timing is indeed affected by distracted attention, but that the pattern of this interference does not match the patterns predicted by the AG theory. Instead, response latency to a timed action was predominantly predicted by distraction around the time of responding, rather than during the interval itself. These results are more in line with an AC account in which distracted attention has the strongest effect around the time an action should have been performed. Distraction from timing during a game of StarCraft2 is not surprising, as players are known to perform a large number of actions per minute. This may vary from about 50 actions per minute in novices to 450 actions per minute in professional players.

The accuracy of timed intervals was operationalized as time between the end of a previous 29‐s interval and a new reaction. Distracted attention was operationalized as the APS measured during a period immediately leading up to the response and a period during which the 29‐s interval was still ongoing. According to AG theory, it should not matter when distraction happens; APS during either period is expected to influence IRT comparably (Zakay & Block, 1997). If attention toward time is decreased because a player is attending to other actions, fewer pulses will reach the accumulator mechanism, irrespective of *when* this distraction occurs. In contrast, the AC theory would predict that increase in IRT is mostly caused by distracted attention during the end of an interval when an action needs to be initiated to check the clock. Based on 28,354 datasets, we did not observe an effect of APS during the interval when we also included the APS just before the next injection was executed. This behavioral signature matches the AC theory instead of the AG theory.

These findings extend the results of Taatgen et al. (2007) in which an analysis of timing accuracy assessed during easy and difficult concurrent tasks led to similar conclusions. Taatgen et al. (2007) hypothesized that temporal units are collected without a gating mechanism, and thus is independent from whether attention is allocated to time or to other tasks. However, following their account, attention is required for checking whether the accumulation of units has crossed a target value. The response behavior of StarCraft2 players is consistent with this explanation; attention is needed when the clock needs to be read out, not during the timing itself. The slower IRTs result from insufficient attentional resources being available to check whether enough time has passed as only if enough attentional resources are available can the clock be actively checked frequently enough to respond in time.

This conclusion is drawn based on data of a well‐trained and highly motivated group of participants in a naturalistic setting. This is an advantage over typical lab‐based studies in which participants are required to do tasks that they are not intrinsically motivated to perform, and that are typically difficult to generalize to real‐world situations (Darlow et al., 2013; Matthews & Meck, 2014). Although comparing the expertise of the players in this study to other domains might be difficult, these players will have had months of “training” in playing the game, and thus have had much more practice with the temporal task than participants in regular lab‐based timing studies.

Importantly, the task has similarities with classic foreperiod experiments (Niemi & Näätänen, 1981; Woodrow, 1914) in which response time to a stimulus was strongly determined by the expectancy of that stimulus. The more frequent a particular foreperiod is experienced, the faster responses become on average (Salet et al., Submitted). Although the field of temporal attention has revealed how temporal regularities are used to adapt our behavior (Nobre & Ede, 2018), the current study contributes to this field by examining this relationship in a dynamic setting in which multitasking is required. Also, the task has similarities with tasks involving differential reinforcement of low rates (DRL), in which participants are rewarded for responding as frequently as possible. A response is only rewarded after a predictable waiting period while a too early response resets the waiting period. Participants in these experiments are able to adjust their responses to their own impreciseness in a near‐optimal way (Balci et al., 2011) in order to maximize the reward. A similar result was found in the current study, thus contributing to earlier findings in the field of decisions‐under‐risk.

Some gaming behavior that is unrelated to timing or attention may partially influence the results of our analyses. For example, expert players describe a habit of keeping their hands “warmed up” during idle moments by rapidly cycling through the routines of selecting their units without issuing commands to them (Huang et al., 2017). However, this behavior is reported to occur predominantly at the onset of a match, before game progress has made Injections possible. Also, as players will typically build multiple hatcheries over the course of a game, multiple intervals might need to be timed concurrently which would influence temporal accuracy (Van Rijn & Taatgen, 2008). However, players counter this challenge by injecting all their hatcheries in one very fast sequence of actions.

This study provides a framework for studying a wide variety of cognitive functions in well‐trained individuals. The richness and availability of replay data from StarCraft2 far exceed any form of laboratory studies. Using data mining of the RTS game StarCraft 2, we contrasted the AG theory and the AC theory in motivated timers. We found support for the view that distracted attention influences timing predominantly when it occurs around the moment a response is required, questioning the continuously modulating role of attention as proposed in most current literature.

### Open Research badges

This article has earned Open Data and Open Materials badges. Data and materials are available at https://osf.io/fwmj7/.
